# Association between quantitative morphological traits and RAPD molecular markers in pomegranate (*Punica granatum* L.)

**DOI:** 10.1002/fsn3.3744

**Published:** 2023-10-08

**Authors:** Ali Khadivi, Akram‐Sadat Hosseini, Fatemeh Kashi

**Affiliations:** ^1^ Department of Horticultural Sciences, Faculty of Agriculture and Natural Resources Arak University Arak Iran; ^2^ Arak University Arak Iran

**Keywords:** fruit; pomegranate, molecular markers, regression analysis

## Abstract

Pomegranate (*Punica granatum* L.) is very important in terms of horticulture and food around the world. The present research aimed to identify the random amplified polymorphic DNA (RAPD) markers associated with morphological traits in pomegranate genotypes. Significant differences were observed among the studied genotypes based on the recorded traits. The 18 RAPD primers produced a total of 154 polymorphic fragments among genotypes. Using multiple regression analysis between each of the morphological traits and 154 RAPD polymorphic bands, RAPD markers associated with each of the morphological traits were identified. In total, 11 markers showed significant correlations with fruit weight, 9 markers with 100‐aril weight, 11 markers with anthocyanin, and 8 markers with total soluble solids. Some markers were associated with more than one morphological trait, showing that the association of a marker with more than one trait can be caused by the pleiotropic effects of quantitative trait loci related to each other in different traits. For instance, the BA6‐1 marker showed positive correlations with fruit weight, fruit crown width, and leaf length. Also, OPG13‐3 and BA6‐10 markers showed positive correlations with total soluble solids and anthocyanin content. The informative markers identified related to morphological characteristics in pomegranate can be a suitable guide to identify the genotypes with valuable fruit traits. Also, these markers can be used in selecting suitable parents for population generation for mapping purposes.

## Graphical abstract

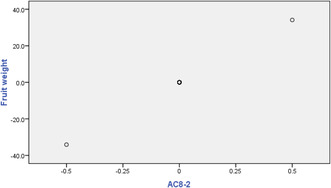

## 1 INTRODUCTION

Pomegranate (*Punica granatum* L.) is very important in terms of horticulture and food in the world, and it is further used in the food industry to produce juice, alcoholic beverages, and seed oil (Holland et al., 2009). Most likely, the origin of commercial pomegranate (*P. granatum*) is Iran, and from there it was taken to areas like India, China, Afghanistan, Pakistan, and the Mediterranean through trade exchanges. Several reports have recorded pomegranate as self‐fertile, cross‐fertile, or self‐fertile/cross‐fertile (Karale et al., 1993). Different parts of pomegranate, including leaves, bark, trunk, root, fruit peel, fruit juice, and seeds, contain effective compounds that can include extensive antioxidant activity in addition to antimicrobial properties (Gil et al., 2000).

During the past years, molecular markers based on DNA have been widely used for various purposes in fruit trees, including sour cherry (Yaman, 2022), apricot (Yaman & Uzun, 2021), walnut (Yildiz et al., 2021), and banana (Pinar et al., 2019). Constant progress in improving plant species breeding depends on the genetic diversity of plants. Therefore, identification and management of this diversity are necessary for breeding programs. In addition, knowledge of genetic diversity makes it easier to manage the protection of plant germplasm (Neale & Savolainen, 2004).

Although mapping based on quantitative trait loci (QTLs) is suitable for tracking genes related to these traits, this process is time‐consuming and laborious (Neale & Savolainen, 2004). To overcome these limitations, it seems appropriate to identify markers dependent on traits through regression. Multiple regression analysis (MRA) based on the relationship between molecular markers, as independent variables, and morphological traits, as dependent variables, is a suitable method to identify trait‐dependent markers. This analysis determines the determination coefficient (*r* ^2^), which shows the relationship between the morphological trait and the molecular marker (Roy et al., 2006). Also, MRA has been used to determine the relationships between morphological characters (Beigi & Khadivi, 2023; Khadivi et al., 2021; Khadivi & Beigi, 2022; Mirheidari et al., 2022; Mirmahdi & Khadivi, 2021; Zandiehvakili & Khadivi, 2021).

The availability of a large number of molecular markers and morphological traits can help to study the regression analysis between these markers and morphological traits. Most of the relational analysis studies based on continuity have made it possible to track different markers related to morphological traits, but often, due to the large distance between the marker and the morphological trait, selection is made with the help of the associated marker, as well as isolation and assimilation. It has made finding the desired gene difficult, and in addition, only a small number of genotypes have been used as parents for gene mapping. In recent years, to overcome this problem, regression analysis between markers and morphological traits has been used, which not only enables the mapping of genes with a higher degree of confidence but also the identification of markers that are on the map. It enables detection based on untraceable linkage (Jugran et al., 2013; Kar et al., 2008).

The molecular markers have been used to determine trait‐related markers in some plants. For instance, the relationship between the data obtained from different molecular techniques and biochemical traits has been investigated in *Morus* spp. (Kar et al., 2008) and *Valeriana jatamansi* (Jugran et al., 2013). Also, the relationship of molecular markers with morphological traits has been investigated in cotton (Shen et al., 2007; Zeng et al., 2009), wheat (Ma et al., 2005), maize (Song et al., 2004), *Morus laevigata* (Chatterjee et al., 2004), sweet cherry (Khadivi‐Khub, 2014), cashew (Timmappaiah et al., 2009), and almond (Khadivi et al., 2023).

The random amplified polymorphic DNA (RAPD) technique is based on the replication of random DNA fragments and does not require knowledge of the DNA sequence of the template. Other advantages of this technique include low cost, simplicity, high speed, and the need for a small amount of DNA, but it has limitations such as low reproducibility due to the random nature of the primers and the sensitivity of the technique to variations in the PCR conditions (Khadivi‐Khub, 2014).

The regression relationship of morphological traits with molecular markers has not been investigated for pomegranate. Therefore, the present research aims to identify RAPD markers associated with important morphological traits in pomegranate genotypes using MRA. The result of this study helps to identify the genes responsible for the emergence of important traits that can be used in breeding programs.

## 2 MATERIALS AND METHODS

### 2.1 Plant material

The present research aimed to identify RAPD markers associated with morphological traits in 12 pomegranate genotypes from the Saveh region in Markazi province, Iran. The selected genotypes were mature (10–12 years old), healthy, and had a full crop. Common orchard management, including irrigation, nutrition, and pest and disease control, was regularly done.

### 2.2 Morphological evaluation

The evaluated morphological traits included those related to the fruit and leaf. Traits were evaluated on 50 fruits of each genotype over two years. A digital caliper was used to measure the dimensions of different organs. The weight of seed, aril, and fruit was measured with a digital scale. A refractometer was used to measure total soluble solids (TSS), and its unit was °Brix. To measure titratable acidity (TA), titration with pH 7 and 0.01 N NAOH was used and its unit was expressed as g/L of citric acid. A pH meter was used to measure pH.

### 2.3 Molecular evaluation

DNA extraction from mature and fresh leaf samples was done using the method of Doyle and Doyle (Doyle & Doyle, 1987). The quantity and quality of the obtained DNA were determined using spectrophotometry at 260 and 280 nm wavelengths and DNA electrophoresis in agarose gel with a concentration of 1.00%, and with their help, the same concentration of DNA (5 ng μL^−1^) was determined. Sterile double‐distilled water was used for dilution. The PCR reaction for the RAPD technique was based on the method described by Khadivi‐Khub (2014). For each reaction mixture, 1 μL of DNA prepared with a concentration of 5 ng μL^−1^ was added to 24 μL of PCR reaction mixture, including dNTPS, PCR buffer, *Taq* DNA polymerase, primer, and MgCl_2_ (Cinagen Company), and sterile double‐distilled water was added. Finally, the volume of the PCR reaction solution reached 25 μL, and the resulting mixture was subjected to the polymerase chain reaction in a Bio‐Rad thermal cycler model I‐Cycler. Thermal cycles include 94°C for 4 min for initial annealing, 35 cycles at 65°C for 30 s for annealing, an extension temperature of 37°C for 1 min, the reproduction temperature of the fragments being 72°C for 2 min, and finally a temperature cycle of 72°C for 5 min to complete the expansion. PCR product electrophoresis was analyzed in agarose gel with a concentration of 1.50%.

### 2.4 Statistical analysis

For fruit traits, means were compared using SAS software version 6 and Duncan's test. For molecular analysis, after performing RAPD tests, to check the genetic polymorphism between genotypes, the presence of a specific band was given a value of 1 and its absence was given 0. The size of amplified bands was estimated by Quantity One software. For each primer, the total number of produced bands, the number of polymorphic bands, and the percentage of polymorphism were calculated.

Stepwise regression analysis was done to trace the relationship between the tested morphological traits as dependent variables and RAPD markers as independent variables using SPSS version. 16. The *r* ^2^ and *β* coefficients were calculated using regression analysis and were investigated for different markers related to traits. The *r* ^2^ is the multiple justified correlation coefficient that is calculated for each marker and indicates the correlation of the marker with the morphological trait. Also, *β* is the standardized regression coefficient, which is calculated using MRA for each trait‐related marker (Kar et al., 2008). Cluster analysis was performed using the Euclidean distance coefficient and Ward's method for morphological data and also using the UPGMA method and Jaccard similarity coefficient for molecular data with PAST software (Hammer et al., 2001).

## 3 RESULTS

### 3.1 Morphological analysis

**FIGURE 1:**
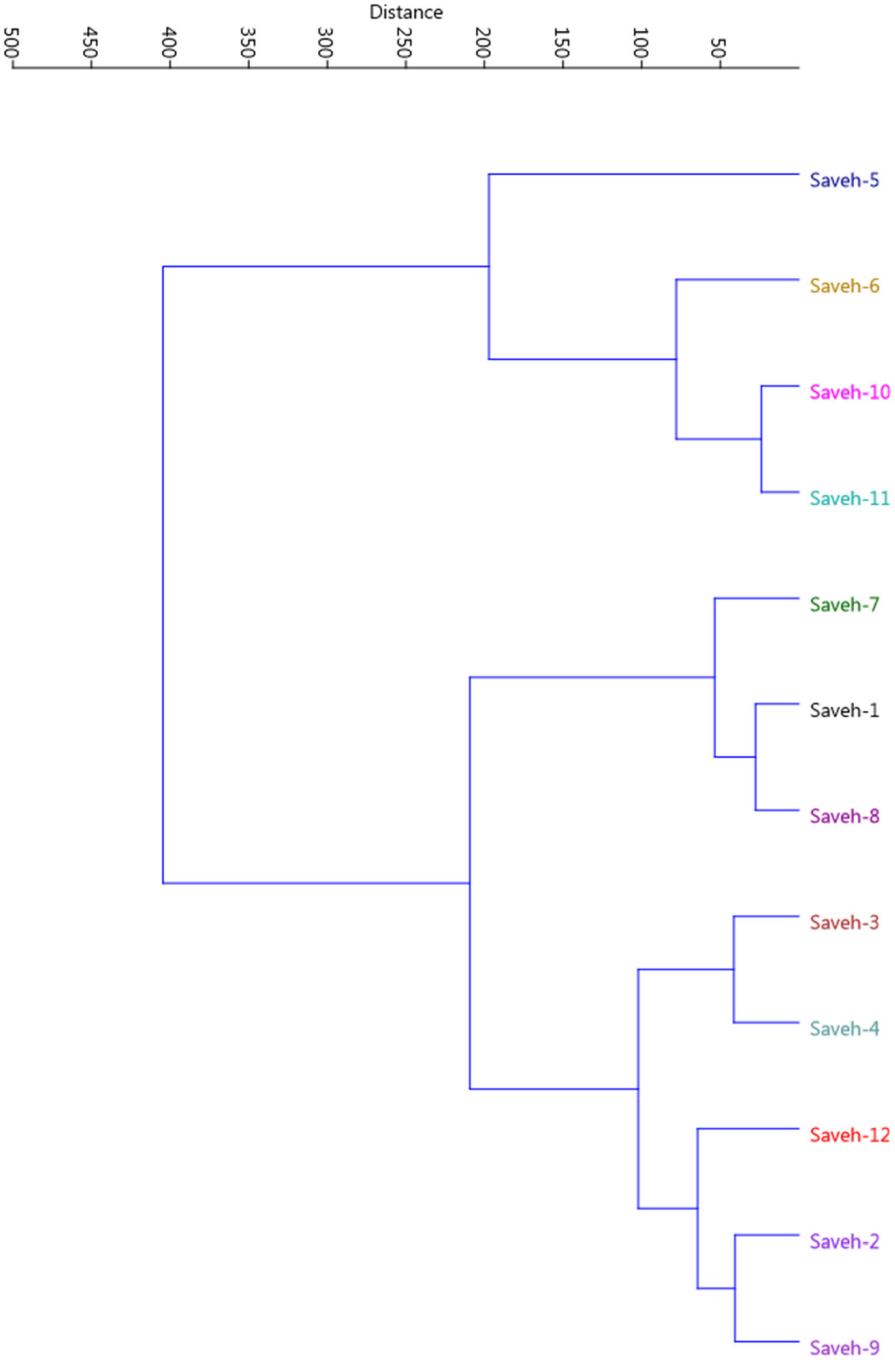
Ward cluster analysis of the studied pomegranate genotypes based on morphological traits using Euclidean distances.

Significant differences were observed among the studied genotypes based on the recorded traits. Fruit weight ranged from 90.12 to 249.46 g. Aril weight per fruit ranged from 31.87 to 164.98 g, while 100‐aril weight varied from 19.01 to 37.91 g. Aril length ranged between 0.92 and 1.17 cm, and aril diameter varied from 0.62 to 0.81 cm. The range of chemical properties was as follows: anthocyanin content: 7.00–174.00 O.D., TSS: 10.60%–15.00%, TA: 0.50%–5.03%, and pH: 2.56–3.45 (Table 1). The dendrogram created through Ward's method and Euclidean distance divided the genotypes into two clusters, which indicates high diversity among the genotypes (Figure 1).

**TABLE 1:** Descriptive statistics for fruit characters among the studied pomegranate genotypes.

No.	Trait	Unit	Min	Max	Mean
1	Fruit weight	g	90.12	249.46	170.92
2	Fruit crown length	cm	1.13	3.36	2.00
3	Fruit crown width	cm	0.09	3.32	1.62
4	100‐aril weight	g	19.01	37.91	28.76
5	Anthocyanin content	O.D. 510 nm	7.00	174.00	46.17
6	Total soluble solids	%	10.60	15.00	12.89
7	Titratable acidity	% Citric acid	0.50	5.03	1.69
8	pH	–	2.56	3.45	3.06
9	Aril weight per fruit	g	31.87	164.98	103.65
10	Peel weight per fruit	g	36.06	121.42	66.90
11	Aril length	cm	0.92	1.17	1.06
12	Aril diameter	cm	0.62	0.81	0.72
13	Leaf length	cm	3.53	7.75	5.30
14	Leaf width	cm	1.08	2.29	1.38
15	Petiole length	cm	0.27	0.71	0.48
16	Fruit peel thickness	cm	0.20	0.41	0.27

### 3.2 Molecular analysis

**FIGURE 2:**
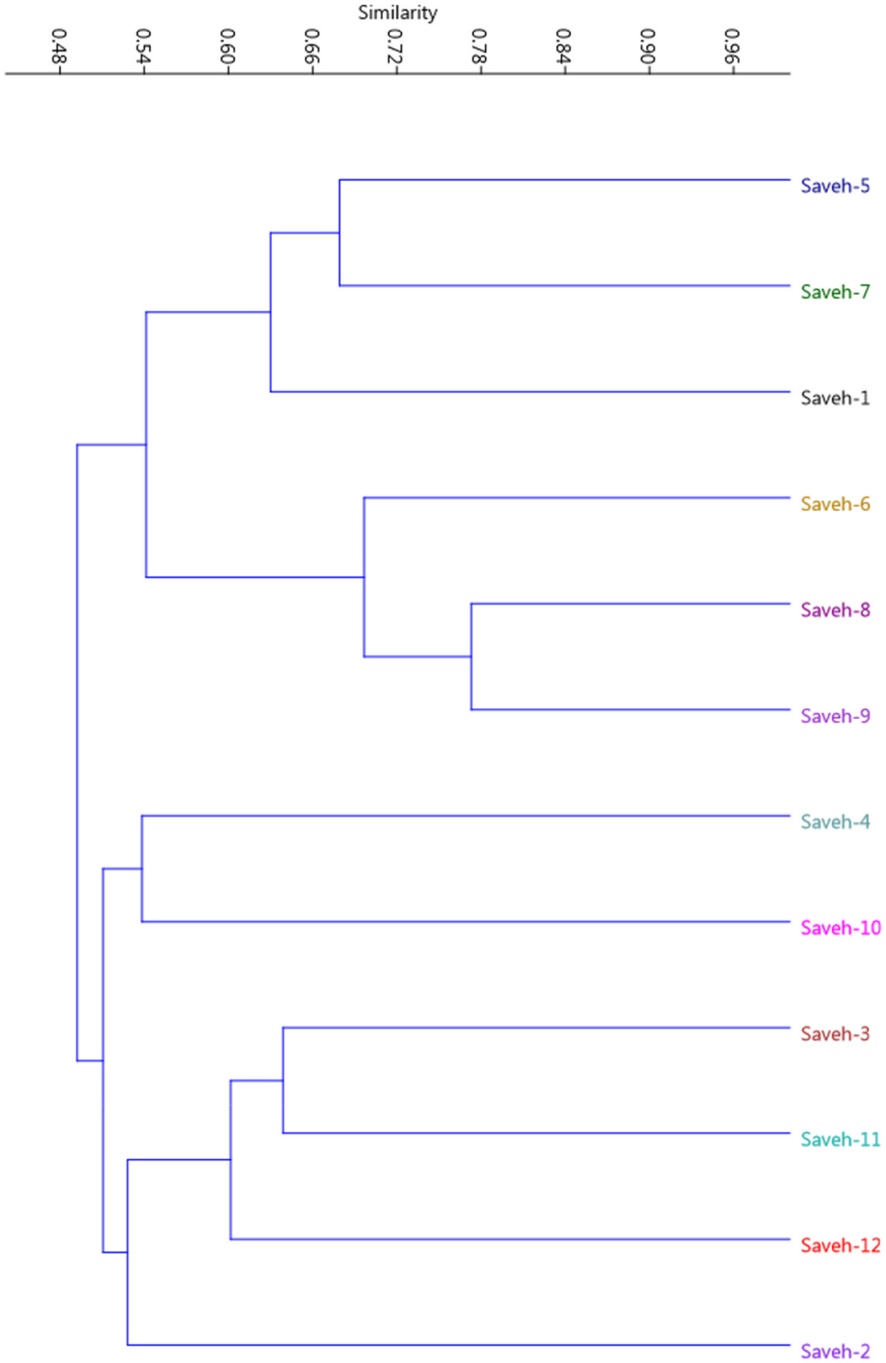
UPGMA cluster analysis of the studied pomegranate genotypes based on RAPD molecular marker.

A total of 18 RAPD primers were selected for PCR reactions and were used for all the genotypes. The 18 primers produced a total of 154 polymorphic fragments among genotypes. Also, the average polymorphism detected by the used primers was equal to 93.46%, which indicated high genetic diversity in the tested germplasm. The dendrogram created through the UPGMA method and Jaccard similarity coefficient divided the genotypes into two clusters, which indicates high diversity among the genotypes (Figure 2).

### 3.3 Association between morphological data and RAPD molecular markers

**FIGURE 3:**
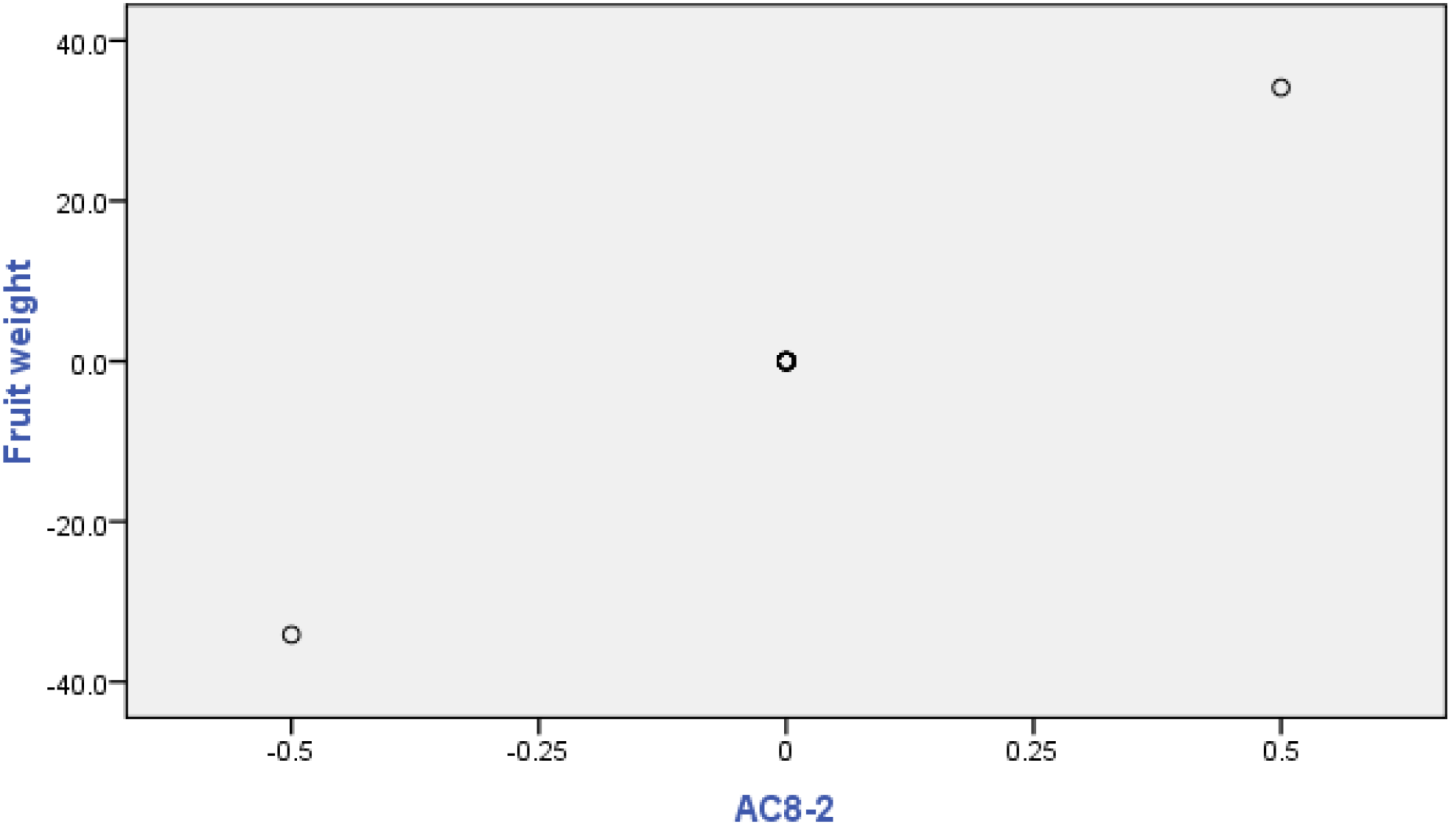
Regression plot for fruit weight with RAPD marker AC8‐2. The *x*‐axis variable is residual from regressing AC8‐2 against the remaining independent variables.

Using MRA between each of the morphological traits with 154 RAPD polymorphic bands, RAPD markers associated with each of the morphological traits were identified. In total, 11 markers showed significant correlations with fruit weight; among them, the AC8‐2 marker showed the highest *β* (Table 2). The association between fruit weight and the AC8‐2 marker is shown in Figure 3. Also, 11 markers showed significant correlations with fruit crown length; among them, the BB13‐1 marker had the highest *β*. In addition, nine markers showed significant correlations with fruit crown width; among them, the OPN8‐4 marker showed the highest *β*. Besides, 9 markers showed significant correlations with 100‐aril weight; among them, the OPG13‐2 showed the highest *β*. Anthocyanin content showed significant correlations with 11 markers; among them, OPG13‐3 marker had the highest *β* (Table 2). TSS showed significant correlations with 8 markers; among them, the OPG13‐3 marker had the highest *β*. Also, 9 markers showed significant correlations with TA; among them, the OPN1‐10 marker showed the highest *β*. The pH showed significant correlations with 9 markers, among them, the OPG11‐5 marker had the highest *β* (Table 2). Aril weight per fruit showed significant correlations with 6 markers; among them, the BA6‐12 marker had the highest *β*. Also, 3 markers showed significant correlations with peel weight per fruit; among them, the BB15‐2 marker showed the highest *β* (Table 2).

**TABLE 2:** The results of MRA between RAPD marker polymorphic bands and morphological traits of the studied pomegranate genotypes.

Trait	RAPD marker	*r*	*r* ^2^	*β*	*t*	*p*
Fruit weight	BB17‐9	.646 a	.42	−.79	–	–
	AC8‐2	.888 b	.79	.64	–	–
	BA6‐1	.964 c	.93	.39	–	–
	OPN16‐5	.984 d	.97	−.25	–	–
	OPN8‐3	.995 e	.99	.19	–	–
	BB3‐1	.998 f	1.00	.09	–	–
	OPN1‐3	1.000 g	1.00	−.06	–	–
	OPG13‐13	1.000 h	1.00	−.03	–	–
	BA5‐15	1.000 i	1.00	.01	–	–
	BA7‐7	1.000 j	1.00	.00	–	–
	OPK10‐4	1.000 k	1.00	.00	–	–
Fruit crown length	BA5‐3	.763 a	.58	−.76	–	–
	BB13‐1	.928 b	.86	.58	–	–
	OPAB4‐11	.982 c	.97	−.29	–	–
	BB11‐1	.997 d	.99	.24	–	–
	BB17‐13	.999 e	1.00	−.09	–	–
	BB13‐4	1.000 f	1.00	−.08	–	–
	OPN16‐2	1.000 g	1.00	−.06	–	–
	BB17‐2	1.000 h	1.00	−.01	–	–
	BB15‐13	1.000 i	1.00	−.01	–	–
	BA6‐12	1.000 j	1.00	.00	–	–
	OPG13‐3	1.000 k	1.00	.00	–	–
Fruit crown width	OPN8‐4	.651 a	.42	1.07	676.06	.00
	BB14‐5	.845 b	.71	−.58	−608.19	.00
	BA5‐8	.951 c	.91	−.76	−368.70	.00
	BA5‐3	.987 d	.98	.38	193.15	.00
	OPN16‐3	.995 e	.99	−.22	−223.93	.00
	BA6‐10	1.000 f	1.00	−.10	−78.12	.00
	AC8‐1	1.000 g	1.00	.02	28.92	.00
	BA6‐12	1.000 h	1.00	−.02	−15.65	.00
	BA6‐1	1.000 i	1.00	−.01	−6.31	.02
100‐aril weight	OPG13‐2	.769 a	.59	.60	304.18	.00
	OPN1‐6	.874 b	.77	−.60	−336.60	.00
	BA5‐14	.952 c	.91	.14	69.93	.00
	BB3‐1	.975 d	.95	−.39	−180.23	.00
	BA5‐11	.992 e	.98	−.35	−144.89	.00
	BA5‐15	.999 f	1.00	.17	90.96	.00
	BB7‐2	1.000 g	1.00	.06	27.18	.00
	BA7‐1	1.000 h	1.00	.04	19.41	.00
	BB7‐8	1.000 i	1.00	−.02	−7.24	.02
Anthocyanin content	OPK10‐6	.807 a	.65	−1.41	–	–
	AC8‐6	.957 b	.92	−.60	–	–
	OPG13‐3	.983 c	.97	.32	–	–
	BA6‐10	.995 d	.99	.16	–	–
	BA7‐1	.999 e	1.00	.10	–	–
	BB15‐4	1.000 f	1.00	.07	–	–
	BB3‐3	1.000 g	1.00	−.03	–	–
	OPN16‐7	1.000 h	1.00	−.01	–	–
	BB13‐5	1.000 i	1.00	.00	–	–
	BB13‐3	1.000 j	1.00	.00	–	–
	OPG11‐3	1.000 k	1.00	.00	–	–
Total soluble solids	BA7‐7	.645 a	.42	.37	–	–
	OPAB4‐2	.884 b	.78	−.64	–	–
	OPG13‐3	.943 c	.89	.42	–	–
	BA6‐10	.980 d	.96	.37	–	–
	AC8‐6	.993 e	.99	.22	–	–
	OPN1‐1	.998 f	1.00	−.14	–	–
	BB15‐2	1.000 g	1.00	.07	–	–
	BB3‐2	1.000 h	1.00	−.03	–	–
Titratable acidity	OPK10‐6	.809 a	.66	−1.07	−1991.00	.00
	BA5‐7	.945 b	.89	−.44	−903.65	.00
	BB13‐5	.991 c	.98	−.33	−277.21	.00
	OPN1‐10	.998 d	1.00	.21	410.63	.00
	BB7‐3	1.000 e	1.00	−.08	−256.99	.00
	OPN16‐5	1.000 f	1.00	.02	41.09	.00
	BB15‐13	1.000 g	1.00	.01	40.42	.00
	BA6‐6	1.000 h	1.00	−.01	−13.37	.01
	OPG11‐2	1.000 i	1.00	.00	6.11	.03
pH	BB7‐1	.715 a	.51	−.83	−39,570,000.00	.00
	OPN16‐6	.902 b	.81	.41	19,810,000.00	.00
	BB17‐9	.963 c	.93	.24	13,220,000.00	.00
	OPG11‐5	.987 d	.97	.46	19,510,000.00	.00
	OPG13‐2	.997 e	1.00	.28	16,780,000.00	.00
	OPN1‐3	.999 f	1.00	−.04	−2,362,000.00	.00
	OPG11‐1	1.000 g	1.00	−.06	−3,716,000.00	.00
	OPG13‐1	1.000 h	1.00	−.05	−2,073,000.00	.00
	OPN1‐9	1.000 i	1.00	−.02	−929,100.00	.00
Aril weight per fruit	BB15‐13	.679 a	.46	−.65	−25.19	.00
	BA6‐12	.935 b	.87	.70	36.40	.00
	OPG13‐13	.971 c	.94	.27	11.24	.00
	BA6‐11	.984 d	.97	−.13	−5.66	.00
	OPAB4‐8	.994 e	.99	−.20	−9.91	.00
	BB17‐2	.999 f	1.00	.16	5.99	.00
Peel weight per fruit	BB17‐6	.855 a	.73	−.80	−16.48	.00
	OPN8‐5	.936 b	.88	−.53	−9.99	.00
	BB15‐2	.991 c	.98	.36	6.80	.00
Aril length	BB17‐3	.619 a	.38	−1.75	–	–
	OPN8‐8	.812 b	.66	.98	–	–
	OPK10‐3	.893 c	.80	−.76	–	–
	BB13‐4	.961 d	.92	.75	–	–
	OPN1‐5	.996 e	.99	.47	–	–
	BB17‐6	.999 f	1.00	.15	–	–
	OPN16‐8	1.000 g	1.00	.09	–	–
	OPK10‐9	1.000 h	1.00	−.01	–	–
Aril diameter	BB7‐1	.775 a	.60	−.94	−32,140,000.00	.00
	OPAB4‐8	.934 b	.87	−.78	−23,880,000.00	.00
	OPAB4‐1	.974 c	.95	.44	13,890,000.00	.00
	OPG11‐3	.986 d	.97	.22	11,480,000.00	.00
	AC8‐3	.996 e	.99	−.25	−12,090,000.00	.00
	BA6‐7	.999 f	1.00	−.18	−4,626,000.00	.00
	BA6‐6	1.000 g	1.00	.07	2,676,000.00	.00
	OPN16‐2	1.000 h	1.00	−.04	−1,813,000.00	.00
	BB15‐4	1.000 i	1.00	−.01	−493,500.00	.00
Leaf length	OPG11‐3	.665 a	.44	−.82	–	–
	OPN1‐9	.885 b	.78	.55	–	–
	OPN1‐6	.964 c	.93	−.55	–	–
	OPN16‐4	.987 d	.98	.19	–	–
	OPN16‐5	.997 e	.99	.16	–	–
	AC8‐3	.999 f	1.00	.06	–	–
	BB15‐7	1.000 g	1.00	.07	–	–
	BA6‐1	1.000 h	1.00	.02	–	–
	OPK10‐9	1.000 i	1.00	−.02	–	–
	OPK10‐3	1.000 j	1.00	.00	–	–
	BB14‐5	1.000 k	1.00	.00	–	–
Leaf width	OPG13‐9	.732 a	.54	.83	634.64	.00
	OPAB4‐8	.947 b	.90	.66	370.68	.00
	BB7‐8	.992 c	.98	−.33	−229.12	.00
	BA5‐7	.997 d	1.00	−.12	−83.91	.00
	OPG13‐10	.999 e	1.00	.04	28.49	.00
	BB17‐13	1.000 f	1.00	−.05	−33.20	.00
	BA5‐11	1.000 g	1.00	.04	20.29	.00
	OPAB4‐1	1.000 h	1.00	−.01	−5.48	.01
Petiole length	OPG13‐13	.743 a	.55	.94	–	–
	OPN16‐3	.870 b	.76	−.55	–	–
	OPG13‐3	.952 c	.91	−.47	–	–
	OPN8‐6	.986 d	.97	.27	–	–
	BA6‐1	.997 e	.99	.18	–	–
	BB14‐2	.999 f	1.00	−.09	–	–
	BA5‐14	1.000 g	1.00	−.06	–	–
	OPAB4‐5	1.000 h	1.00	.02	–	–
	OPK10‐2	1.000 i	1.00	−.02	–	–
Fruit peel thickness	OPAB4‐8	.747 a	.56	.55	28,790,000.00	.00
	OPG13‐9	.887 b	.79	.31	25,670,000.00	.00
	OPG13‐1	.961 c	.92	−.40	−36,040,000.00	.00
	BB11‐5	.983 d	.97	.39	23,000,000.00	.00
	BA5‐5	.996 e	.99	−.39	−21,620,000.00	.00
	BA5‐11	.999 f	1.00	.18	14,300,000.00	.00
	OPK10‐4	1.000 g	1.00	−.07	−4,079,000.00	.00
	BB7‐3	1.000 h	1.00	.02	1,644,000.00	.00
	OPAB4‐2	1.000 i	1.00	.02	750,500.00	.00

*Note*: For each trait, the values in a column with different alphabetical letter are significantly different (*p* 〈 .05).

Aril length showed significant correlations with 8 markers; among them, the OPN8‐8 marker had the highest *β*. Also, 9 markers showed significant correlations with aril diameter; among them, the OPAB4‐1 marker showed the highest *β*. Leaf length showed significant correlations with 11 markers; among them, the OPN1‐9 marker had the highest *β* (Table 2). Leaf width showed significant correlations with 8 markers; among them, the OPG13‐9 marker had the highest *β*. Also, 9 markers showed significant correlations with petiole length; among them, the OPG13‐13 marker showed the highest *β*. Fruit peel thickness showed significant correlations with 9 markers; among them, the OPAB4‐8 marker had the highest *β* (Table 2).

## 4 DISCUSSION

The studied pomegranate genotypes showed considerable variation in terms of morphological characterizations and molecular aspects, and as a result of this high diversity, the relationship between molecular markers and morphological traits can be traced through MRA. Some of the fragments produced by RAPD markers showed significant correlations with the morphological traits. The location of these markers inside the genome is probably the region of the genome that encodes the genes related to the desired fruit traits (Virk et al., 1996). With each trait, several correlated markers were detected, and the marker with the highest *r* ^2^ was considered the most effective marker in coding that trait (Kar et al., 2008). Some markers were associated with more than one morphological trait, showing that the association of a marker with more than one trait can be caused by the pleiotropic effects of QTLs related to each other in different traits (Culp et al., 1979; Meredith & Bridge, 1971), but for more information from this relationship, creating a diverging population and mapping its linkage can be useful (Culp et al., 1979). The pleiotropic effect occurs when a gene can have an effect on the occurrence of several traits at the same time. Also, QTLs related to each other that control different traits can lead to the creation of a single marker that is correlated with more than one trait (Meredith & Bridge, 1971). For instance, the BA6‐1 marker showed positive correlations with fruit weight, fruit crown width, and leaf length. Significant positive correlations between the above characters have been previously reported in pomegranate (Khadivi et al., 2018, 2020; Khadivi & Arab, 2021). Khadivi et al. (2023) reported that some polymorphic fragments of ISSR and RAPD showed significant correlations with the fruit traits measured in almonds.

Here, the BA7‐7 marker showed a positive correlation with TSS and a negative correlation with TA. Significant negative correlations between the above characters have been previously reported in pomegranate (Khadivi et al., 2018, 2020; Khadivi & Arab, 2021). OPG13‐3 and BA6‐10 markers showed positive correlations with TSS and anthocyanin content. Significant negative correlations between the above characters have been previously reported in pomegranate (Khadivi et al., 2018, 2020; Khadivi & Arab, 2021).

The MRA is a suitable and quick method to find the relationship between traits and markers (Yao & Mehlenbacher, 2000). The markers identified in this study that have shown significant correlations with fruit traits can be used in MAS breeding programs. The obvious advantage of this analysis is that this method can track QTL. Also, it requires less time and cost (Yao & Mehlenbacher, 2000) and does not need to form a population for mapping (Virk et al., 1996).

The initial selection of desirable traits related to fruit and flowers requires the growth of the plants and their entry into the maturity stage. In other words, the fruit trees must go through a long juvenile stage and enter the fruiting stage so that these traits can be examined and the plants with desirable traits in terms of flowers and fruits can be found (Virk et al., 1996). However, by tracking the markers related to these traits, there is no need for the plants to enter the maturity stage. In other words, for woody plants, such as fruit trees, with a long juvenile period, it is difficult to select the superior progeny in terms of important flower and fruit traits, but by identifying the trait‐dependent markers (MAS), it is possible to identify and select the superior progeny in the early stages of their growth (Virk et al., 1996). The working method is that polymorphic DNA fragments identified as informative markers for the trait under study, such as traits related to fruit, can be separated from the gel and cloned, then, the identified sequence was aligned with the existing sequences in the NCBI database and the candidate genes that have a high similarity to the desired informative markers were identified. It is also possible to design primers for different molecular markers from the obtained sequence and use them in breeding programs through trait‐dependent marker (MAS) selection (Yao & Mehlenbacher, 2000).

The identification of molecular markers related to the main genes controlling the desired traits has been done in recent years by creating differentiating populations such as F1 in heterozygous plants and F2, RIL, and DH in homozygous plants. Some of these markers have been used to perform breeding programs, but the unavailability of diverging populations for mapping, the lack of sufficient time, and the lack of sufficient correlation between morphological traits and molecular markers are among the most important limitations in the field of identifying markers related to morphological traits, but performing MRA lacks these limitations (Miletic et al., 2005).

## 5 CONCLUSIONS

The informative markers identified related to morphological characteristics in pomegranate can be a suitable guide to identify the genotypes with valuable fruit traits. In breeding programs, choosing quality plant materials usually takes a lot of time and cost. However, informative markers identified can be useful in selecting superior genotypes, especially when information about their genetic basis, such as a linkage map, is not available. Also, these markers can be used in selecting suitable parents for population generation for mapping purposes. Based on the ideal values of commercial characteristics of pomegranate, such as fruit weight, aril weight, aril color, anthocyanin content, and TSS, three genotypes, including Saveh‐5, Saveh‐7, and Saveh‐8, were promising and thus could be directly cultivated in the orchards and used in breeding programs.

## AUTHOR CONTRIBUTIONS

**Ali Khadivi:** Formal analysis (lead); investigation (equal); methodology (lead); supervision (lead); writing – original draft (lead); writing – review and editing (lead). **Akram‐Sadat Hosseini:** Investigation (equal). **Fatemeh Kashi:** Investigation (equal).

## CONFLICT OF INTEREST STATEMENT

None.

## Data Availability

The data that support the findings of this study are available from the corresponding author upon reasonable request.
